# Bacteria in tree bark are hungry for methane

**DOI:** 10.1038/s42003-021-02264-1

**Published:** 2021-06-25

**Authors:** George Andrew S. Inglis

**Affiliations:** Communications Biology, https://www.nature.com/commsbio/

## Abstract

While reforestation efforts are important in limiting the progression of climate change, tree stems are known to emit the potent greenhouse gas, methane. Luke Jeffrey and colleagues recently discovered that methanotrophic bacteria colonize the bark of the common lowland tree, *Melaleuca quinquenervia*, and significantly reduce its methane emissions. Their results expand the known pool of habitats for methanotrophic bacteria and suggest that these bark-dwelling taxa may be a future target for limiting methane emissions from trees.

**Figure:**
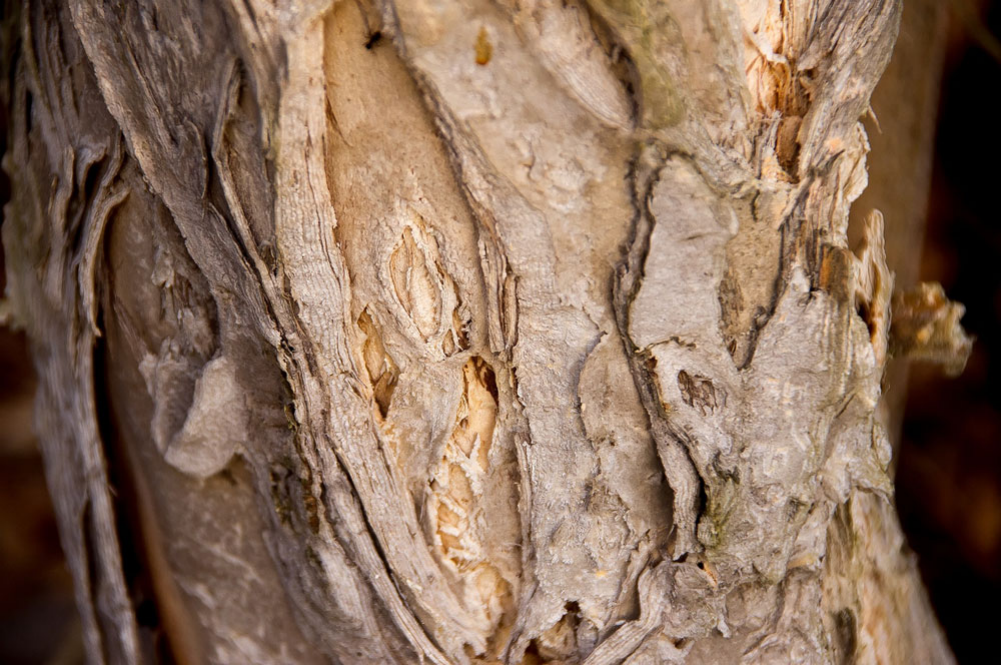
Pixabay

Earth’s forests are one of its most important carbon sinks, environments that can absorb and sequester atmospheric carbon dioxide. While the act of planting trees has become a common practice in the fight against climate change, tree stems also produce methane, a related greenhouse gas that is even more effective than carbon dioxide in warming the atmosphere. Given the importance of balancing reforestation efforts with limiting methane emissions, it is critical to identify potential methane sinks for trees.

Methane-oxidising bacteria (MOB) are known to act as methane sinks in soil and water by utilizing methane as an energy source. A recent study^1^ from Luke Jeffrey et al. at Southern Cross University discovered that MOB are also present in the bark microbiome of *Melaleuca quinquenervia*, a common paperbark tree native to Australia. After gathering samples of *M. quinquenervia* from three forests, the authors identified that these bark swatches were enriched with distinct MOB compared to sediment or water isolates taken from the same locations. Furthermore, they observed a significant association between the abundance of MOB in bark samples and the rate of in vitro methane uptake, suggesting these bark-dwelling microbes were actively oxidising methane. Finally, the authors measured field methane emissions in *M. quinquenervia* which had been treated with difluoromethane, a known inhibitor of methanotrophy in MOB. They observed that application of difluoromethane led to a significant increase in methane levels compared to baseline or control conditions, indicating that MOB may normally limit methane emissions from tree stems.

Altogether, these results suggest that MOB in *M. quinquenervia* bark act as a natural sink to limit methane emissions. While it is currently unclear whether MOB perform a similar role in other species, this study represents an important first step in identifying potential methane sinks for trees.
